# Relating stability of individual dynamical networks to change in psychopathology

**DOI:** 10.1371/journal.pone.0293200

**Published:** 2023-11-09

**Authors:** Sara van der Tuin, Ria H. A. Hoekstra, Sanne H. Booij, Albertine J. Oldehinkel, Klaas J. Wardenaar, David van den Berg, Denny Borsboom, Johanna T. W. Wigman

**Affiliations:** 1 University Medical Center Groningen, Department of Psychiatry, Interdisciplinary Centre Psychopathology and Emotion regulation, University of Groningen, Groningen, The Netherlands; 2 Department of Psychological Methods, University of Amsterdam, Amsterdam, The Netherlands; 3 Center for Integrative Psychiatry, Lentis, Groningen, The Netherlands; 4 Department of Clinical Psychology, Amsterdam Public Health Research Institute, Vrije Universiteit, Amsterdam, The Netherlands; 5 Department of Psychosis Research, Parnassia Psychiatric Institute, The Hague, The Netherlands; UCL University College: UCL Erhvervsakademi og Professionshojskole, DENMARK

## Abstract

One hypothesis flowing from the network theory of psychopathology is that symptom network structure is associated with psychopathology severity and in turn, one may expect that individual network structure changes with the level of psychopathology severity. However, this expectation has rarely been addressed directly. This study aims to examine (1) the stability of individual contemporaneous symptom networks over a one-year period and (2) whether network stability is associated with a change in psychopathology. We used daily diary data of n = 66 individuals, located along the psychosis severity continuum, from two separate 90-day periods, one year apart (t = 180). Based on the newly developed *Individual Network Invariance Test* (INIT) to assess symptom-network stability, participants were divided into two groups with stable and unstable networks and we tested whether these groups differed in their absolute change in psychopathology severity. The majority of the sample (n = 51, 77.3%) showed a stable network over time while most individuals showed a decrease in psychopathological severity. We found no significant association between a change in psychopathology severity and individual network stability. Our results call for further critical evaluation of the association between networks and psychopathology to optimize the implementation of clinical applications based on current methods.

## Introduction

The development of psychopathology involves complex interactions between processes at different levels and time scales [1, 2]. Understanding this complex development might be advanced by viewing psychopathology from a network perspective. The network perspective conceptualizes mental disorders as the result of the direct interplay between symptoms that can trigger and maintain each other [3]. This interplay can be visualized in a network, in which symptoms are represented as nodes, and statistical relationships between these symptoms are represented as edges [4]. The network approach has rapidly gained substantial influence in the field of psychiatry [3, 5–7]. Networks of psychopathological symptoms can be modeled in many ways and a plethora of studies have discussed the principles, applications, merits and drawbacks of a network approach to psychopathology [3–6]. Symptom networks have been estimated from cross-sectional and, increasingly, longitudinal, and time-series data. A critical advantage of the latter is that symptom networks can be constructed per individual by modeling the immediate associations between symptoms using multiple time points—creating the opportunity to gain insight into the dynamic interplay of symptoms at an individual level [8].

One of the main hypotheses flowing from the network theory of psychopathology is that the network structure is associated with the level of psychopathology severity. The symptom structure in a strongly connected network is thought to be self-sustaining: one small perturbation to the network can activate all symptoms in the network, posing a vulnerability to (more) severe psychopathology. On the other hand, when connections between symptoms in such a network are weak, perturbations to the network may have a limited effect, and the system is thought to return to its initial healthy state quickly [3]. Several studies showed that more strongly connected symptom networks are related to more severe and persisting psychopathology [9–11]. Using a multilevel-based estimation technique, de Vos et al., [12] found an overall higher network density within individuals suffering from MDD compared to healthy individuals. However, when taking an individual-level approach using sparse vector autoregression techniques on the same data, they found more and stronger associations in the population network of healthy individuals compared to the network of individuals suffering from major depressive disorder (MDD). In addition, they found no relation between individual network density and psychopathology. This demonstrates that the relation between network structure and psychopathology in longitudinal settings is complex and sensitive to the type of analysis and underline the necessity of further research in order to investigate the relation between (individual) network structure and psychopathology over time.

So far, research on the association between network-structure and psychopathology has mainly looked at the association between longitudinal network structure and cross-sectionally measured psychopathology status/severity. However, given the hypothesized association between symptom-network structure and psychopathology, one may expect that psychopathological severity *changes* alongside the structure of a symptom network: for example, when a symptom network becomes less strongly connected, people would be expected to improve in mental health, and people who develop a more strongly connected symptom network structure are expected to worsen in mental health once their symptom network is activated. This hypothesis has been implicitly assumed often in studies that use network models in relation to clinical applications, where interventions based on network structure are being developed to improve treatment [13, 14]. To the best of our knowledge, it has not yet been investigated how stable individual symptom networks are over time and whether changes in network structure are indeed associated with changes in psychopathology at the individual level. This knowledge could help in the development of such interventions.

As a first steppingstone to investigate the relation between individual network structure and psychopathology severity, this study aimed to examine (1) the stability of individual symptom networks over a period of 1 year and (2) whether symptom-network stability was associated with psychopathology during this time period. There is no clear consensus definition of stability as it is a complex concept. Similar to many other studies, the way that we define stability is based on our statistical approach. Concretely this means that we considered networks stable over time when a single network structure fits the two time periods better in terms of model fit than two different network structures. For a more detailed explanation of stability as interpreted in this paper, see the methods section. The study was performed on a convenience sample of young adult individuals located along the psychosis severity continuum who were considered at increased psychometric or clinical risk for psychosis [15]. We compared two separate periods of diary assessments one year apart, each spanning 90 days of daily symptom reports. We focused solely on contemporaneous symptom networks, which show the associations between symptoms that occur within days (in contrast to temporal symptom networks that show associations between-days) [4]. In accordance with the hypothesis from the network theory of psychopathology, we expected network stability to be associated with psychopathology severity.

## Materials and methods

### Participants and study design

Data came from the Mapping Individual Routes of Risk and Resilience (Mirorr) study [15, 16]. Participants were individuals located along the psychosis severity continuum who were considered at increased psychometric or clinical risk for psychosis (N = 96, age 18–35 years). Participants were assessed during two daily diary periods of 90 days each (one at baseline and one at 1-year follow-up), as well as with cross-sectional questionnaires at baseline and three-yearly follow-up (T0-T3) measurements on mental health and functioning. The sample consisted of N = 25 individuals from the general population who were at increased psychometric risk for psychosis. These were the 25% highest scoring individuals on a questionnaire assessing psychotic experiences (Community Assessment for Psychic Experiences; CAPE) [17]. The other N = 71 individuals were recruited from mental health care institutions in the Netherlands and thus were in clinical care at the moment of inclusion. As the sample consisted of individuals who are *at risk* for psychosis, individuals with a history of, or current psychotic episode, were excluded. The participants who were recruited from mental health care institutions presented with a broad range of mental disorders, but the majority was diagnosed with depression and/or anxiety. This high level of comorbidity and specifically of mood disorders is reflective of the nature of individuals at UHR for psychosis [18].

Inclusion criteria were: (1) age between 18 and 35 years, (2) ability to read and speak Dutch fluently, (3) being capable of following the research procedures, and (4) providing informed consent. Exclusion criteria were: (1) history of or current psychotic episode according to the Diagnostic and Statistical Manual of Mental Disorders 4 (DSM-4) criteria, (2) significant hearing or visual problem impairments, and (3) pregnancy. We refer the interested reader to Booij et al., [15] for a more detailed description of the design and procedure of the Mirorr study.

The study was approved by the medical ethical committee of the University Medical Centre Groningen, Groningen, the Netherlands (registration number MEC no. 2015/159, ABR no. NL52974.042.15). The study was conducted in accordance with the Helsinki Declaration. All participants provided written informed consent.

For the current paper, data from two assessment waves were used: baseline (T0) and follow-up after one year (T1). Both waves contained a 90-day diary study in which several psychopathological symptoms, thoughts, emotions, and behaviors were assessed daily via the participants’ smartphones. To assess symptom-network stability over time, we only included participants who completed diary data at both waves, resulting in a sample of n = 66 individuals. Individuals in this final sample were slightly older than the dropouts (mean age_completers_ = 25.5, sd = 4.49, mean age_dropouts_ = 23.1, sd = 3.07, *p*_difference_ = .03), and did not differ on gender, education and psychopathology.

### Instruments

#### Diary items

The diary questionnaire contained 80 items on a broad range of feelings and emotions, functioning, and behaviors. All items were scored on a 100-point Visual Analogue Scale (VAS) ranging from ‘not at all’ to ‘very much’, and were completed once a day in the evening. Procedures were identical at T0 and T1. We limited the number of variables in the analysis to ensure adequate estimation accuracy and power by computing domain scores through averaging the scores of the items related to the same domain [19]. In line with previous work on these data [20], five domains of psychopathology: irritation, stress, depression, psychosis, anxiety, and one confidence domain were chosen for the current study, which were based on 16 individual items (see S1 Table for item allocation to the domains). We focused specifically on transdiagnostic domains as the early clinical stages for psychosis are characterized by a transdiagnostic and diffuse nature [18]. Domain structure was chosen based on theoretical grounds, i.e., based on which items typically cluster together, and then assessed psychometrically by checking the composite reliability scores. Thus, for all domains, we calculated composite reliability scores taking the multilevel structure into account [21] through the R-package ‘multilevelTools’ [22], resulting in within-person omega’s ranging between .60 and .86 and between-person omega’s ranging between .83 and .99 based on our final sample of n = 66 individuals.

#### Psychopathology questionnaire

As a measure of psychopathology, the Dutch version of the Symptom Checklist Revised (SCL-90-R; [23]) was used. The SCL-90-R measures general psychopathology over the past week, with 90 items scored on a 5-point Likert scale. The SCL-90-R has high reliability (ω = .98; [24]).

### Statistical analyses

All analyses were performed in R version 4.1.0 [25], and alpha *p* < .05 was used as the inference criterion.

#### Descriptive analyses

Descriptive statistics on age, gender, education, general psychopathology, and the five diary domains for the total sample were calculated. Mean psychopathology severity (SCL-90-R) was compared between T0 and T1 by means of a paired t-test. For each of the diary domains, the within-person median over the 90 days was calculated, and then averaged across the total sample. The scores on the diary domains between T0 and T1 were compared with multilevel models (one per domain), as the data was nested within persons, using the R-package ‘nlme’ [26]. All multilevel models had time-point (T0 versus T1) as the fixed effect, random intercepts for subjects, and random slopes for time (the 90 measurements) and time-point.

#### Preprocessing steps

Before constructing symptom networks, (1) missing data were imputed for each individual using exponential moving average, (2) data were transformed with a nonparanormal transformation [27], and (3) de-trended per individual (for more information, see S1 File). This de-trending led to a situation where only model deviations around the individual’s trend were analyzed, so that dynamics in the networks reflect the relation between the deviations from the trend of each domain score. Thus, networks should not be interpreted as reflecting the degree to which stable increases in one symptom domain are associated with stable increases in another domain, but rather as reflecting the degree to which changes in one variable are associated with changes in another variable.

#### Symptom network construction

Individual symptom networks were estimated for each individual at both T0 and T1 separately as graphical Vector Autoregressive models (gVAR) in R using version 0.10 of the ‘psychonetrics’ package [28]. In psychonetrics, a fully connected network model is first estimated. After this, several model selection techniques can be applied to obtain sparse network structures in which only some edges are included. In this paper, we use significance pruning at alpha = 0.05 to obtain sparse individual network structures. By pruning the fully connected network model, edges are excluded based on the set significance level. Next, the network structure is re-estimated with those edges fixed to zero. This re-estimation ensures that the edge weight estimates of the included edges are based on the final model. This leads to more accurate edge weight estimations [29]. The gVAR model assumes stationarity, which means that the time series have time invariant expected values, variances and covariances. Estimating gVAR networks based on longitudinal individual data results in the estimation of a directed, temporal network (based on VAR coefficients) and a contemporaneous network (based on VAR residuals). The temporal network shows the relationship between two variables from the previous measurement occasion onto the next measurement occasion while controlling for the temporal effect of all other variables. The contemporaneous network reflects the unique bidirectional associations among variables that occur after time effects have been taking into account. In previous work, we found that most symptom associations in this dataset occur within days (i.e., contemporaneous) rather than between days (i.e., temporal) [20, 30]. This is in line with other research on diary studies that assess mental states once a day [31]. In addition, assessing the stability of temporal networks with little to no edges has proven to yield limited results [32]. Therefore, for this study, we only interpreted the individual contemporaneous networks. We refer the interested reader to Epskamp et al. [4] for an in-depth discussion on the differences between temporal and contemporaneous networks and their potential in clinical research.

#### Symptom network inspection

To gain more insight into symptom network structures at both time points, we assessed which edges between nodes were estimated in the pruned network structures at T0 and T1, both between- and within-person, by respectively calculating the percentage of edges estimated at T0 and T1 across individuals, and calculating the percentage of individuals for whom an edge was estimated similar at both T0 and T1.

#### Symptom network stability

To test the stability of symptom networks of individuals over time, the *Individual Network Invariance Test* (INIT) was used. INIT makes use of idiographic network estimation techniques as implemented in the ‘psychonetrics’ R package [28]. Psychonetrics estimates a GVAR using Full Information Likelihood (FIML) estimation. This results in the estimation of a saturated network model (i.e., a network in which all edges are estimated to be non-zero) after which several pruning methodologies can be applied to remove edges from the network structure. To make the comparison between network structures more straightforward and stability outcomes not dependent on the pruning technique, we performed INIT on unpruned network structures. INIT estimates a model in which the edge weights of both network structures (at T0 and T1) for one individual are estimated freely, i.e., no constraints are placed between an individual’s network at T0 and T1. In addition, a model is estimated in which the edge weights for both networks (at T0 and T1) of the individual are constrained to be equal, i.e., equality constraints are placed between an individual’s network structure at T0 and T1. INIT determines which of these two models fits best given the data by computing and comparing a model fit index for both models. Model fit is evaluated according to the Akaike Information Criterion (AIC). The model with the lowest AIC is the best fitting model.

Simulation studies showed the AIC fit measure performs best compared to the *χ*^2^ difference test and the Bayesian Information Criteria (BIC) when equality constraints are placed on unpruned individual network structure. Taking the AIC as an indication for model fit, INIT is expected to pick up instability in the network structure with ~100 or more time points per individual. For more details on the performance of INIT see S2 File. INIT is implemented in the ‘INIT’ package in R^1^. As a sensitivity analysis, we also performed INIT on pruned network structures and calculated correlation coefficients between individual network structures at T0 and T1, see S2 File.

Using the above-described approach, we determined for each person whether their symptom networks at T0 and T1 were similar or different over time. Those who had similar symptom networks at T0 and T1 were assigned to the “stable” group and those who had different symptom networks at T0 and T1 were assigned to the “unstable” group. Group membership resulting from INIT was used for further analyses.

#### Descriptive comparison of INIT groups

The stable and unstable groups were compared on age (t-test), gender (Fisher’s exact test), and education (Fisher’s exact test). Psychopathology severity (t-test) and the scores on the six diary domains (multilevel models) were compared between the groups at T0 and T1. As both psychopathology and the diary domains were measured at T0 and T1, we performed extra tests to assess changes from T0 to T1. For a difference in psychopathology, we assessed whether there was a significant change between T0 and T1 for the stable and unstable groups separately by means of a paired t-tests. For differences in diary domains, we assessed whether there was a significant change from T0 to T1 per group separately using multilevel models.

#### The association between network stability and change in psychopathology severity

To assess whether a change in network structure was associated with a change in psychopathology, we tested whether absolute change scores of psychopathology severity differed per INIT group through an independent samples t-test.

#### Power

Previous simulation studies regarding estimating idiographic network structures showed that we have sufficient power to reliably estimate idiographic networks with 90 measurements per person [19]. We conducted a simulation study to test the power to detect instability in individual network models using INIT, see S3 File. Results of this simulation study indicated with six variables and 90 time points per individual that INIT is able to pick up instability between network structures.

^1^You can find INIT on https://github.com/RiaHoekstra/INIT

## Results

### Demographics

For information on the full sample demographics and differences in means of psychopathology as measured with the SCL-90, as well as mean difference for diary domains at T0 and T1, see Table 1.

**Table 1:** Demographics, psychopathology and diary domains for T0 and T1 for full sample.

**Demographics**		
Age (mean, SD)	25.5 (4.49)	
Gender (female)	53 (80%)	
Education		
Low	10 (15%)	
Middle	36 (54%)	
High	20 (30%)	
**Psychopathology**	**T0**	**T1**
	*Mean (SD)*	
SCL-90*	189.24 (57.66)	160.30 (47.40)
**Diary domains**		
	*Median (IQR)*	
Irritation*	19.3 (28.7)	14.5 (21.1)
Stress*	31.1 (25.8)	25.8 (31.9)
Depression*	25.6 (28.1)	22.3 (20.8)
Psychosis*	7.6 (9.9)	6.4 (9.3)
Anxiety*	17.9 (28.3)	12.7 (23.3)
Confidence*	47.9 (22.3)	50.1 (18.8)

*Note*.

*Significant difference between measures on T0 and T1, *p* < .05

### Symptom network structure

The number of times an edge was detected across individuals at T0 and T1, as well as the number of individuals for whom an edge was estimated similar at both T0 and T1 was relatively stable, see Table 2.

**Table 2:** Frequency and percentage of estimated edges at T0 and T1, and frequency and percentage of estimated edges at both T0 & T1, for the full sample.

Edge	T0		T1		T0 & T1	
	Frequency	Percent	Frequency	Percent	Frequency	Percent
Depression-confidence	59	89.4%	60	90.9%	55	83.3%
Stress-anxiety	44	66.7%	39	59.1%	43	65.0%
Irritation-depression	31	47.0%	30	45.5%	35	53.0%
Depression-anxiety	25	37.9%	29	43.9%	54	81.8%
Irritation-stress	20	30.3%	27	40.9%	33	50.0%
Irritation-psychosis	20	30.3%	26	39.4%	40	60.6%
Psychosis-anxiety	19	28.8%	28	42.4%	43	65.0%
Stress-depression	15	22.7%	10	15.2%	43	65.0%
Psychosis-confidence	15	22.7%	9	13.6%	52	78.8%
Depression-psychosis	14	21.2%	15	22.7%	43	65.2%
Stress-confidence	13	19.7%	18	27.3%	39	59.1%
Irritation-confidence	12	18.2%	4	6.1%	50	75.8%
Stress-psychosis	11	16.7%	19	28.8%	44	66.7%
Irritation-anxiety	10	15.2%	13	19.7%	45	68.2%
Anxiety-confidence	10	15.2%	8	12.2%	52	78.8%

*Note*. N = 66

### Symptom network stability

Application of INIT revealed, based on the AIC, that for 51 out of the 66 individuals the model with equality constraints showed the best model fit. These 51 individuals were labeled as the ‘stable group’. Thus, the symptom networks of these individuals were considered similar in structure (i.e., edge weights) at both time points. For the other 15 individuals, the model without equality constraints fit the data better; these individuals were labeled the ‘unstable group’. Thus, the symptom networks of these individuals were considered different in structure at both time points.

### Demographic comparison of INIT groups

The stable and unstable groups did not differ significantly in age, gender and education level. In addition, we found no significant group differences in psychopathology severity, nor in diary domain scores. For more details on demographics by group, see Table 3. We did find a significant difference over time within both groups: the difference in psychopathology severity between T0 and T1 was significant for both the stable group (t = 4.6, *p* < .01) and the unstable group (t = 3.9, *p* < .01). Multilevel models showed that out of the five domain scores, depression, psychosis, anxiety, and confidence differed significantly between T0 and T1 for the stable group, and all five domain scores differed significantly between T0 and T1 for the unstable group.

**Table 3:** Demographics, psychopathology, and diary domains for the stable and unstable group.

**Demographics**	**Stable group**		**Unstable group**	
	n = 51		n = 15	
	*Mean (SD)*		*Mean (SD)*	
Age	25.7 (4.4)		25.4 (4.6)	
Gender	*Frequency (%)*		*Frequency (%)*	
Female	41 (80%)		12 (80%)	
Male	10 (20%)		3 (20%)	
Education				
Low	7 (14%)		3 (20%)	
Middle	26 (51%)		10 (66%)	
High	18 (35%)		2 (13%)	
**Psychopathology**	**T0**	**T1**	**T0**	**T1**
	*Mean (SD)*			
SCL-90	185.2 (56.0)	159.9 (44.9)	203.1 (63.1)	161.8 (56.9)
**Diary domains**				
	*Median (IQR)*			
Irritation	19.2 (30.2)	14.3 (23.2)	19.5 (24.0)	14.7 (16.2)
Stress	29.9 (28.1)	25.6 (32.2)	45.5 (20.8)	28.2 (22.1)
Depression	21.4 (27.2)	21.3 (23.1)	37.4 (26.8)	25.3 (10.7)
Psychosis	7.5 (8.2)	6.4 (9.2)	7.9 (10.2)	6.4 (8.9)
Anxiety	18.3 (24.8)	12.8 (23.3)	14.8 (46.1)	9.2 (13.4)
Confidence	49.3 (20.6)	49.5 (16.9)	41.4 (29.6)	52.2 (28.3)

### The association between network stability and change in psychopathology

A two-sample t-test showed that the stable and the unstable group did not differ significantly in their absolute psychopathology-severity change scores (mean(sd)_stable_ = 38.96 (25.45), mean(sd)_unstable_ = 42.87 (39.13), t = .39, *p* = .70).

## Discussion

This study aimed to investigate the association of the stability of individual symptom networks over time with changes in psychopathology in a convenience sample of individuals along the psychosis severity continuum who were considered at increased psychometric or clinical risk for psychosis. We found that contemporaneous symptom networks were stable between two time-points one year apart for the majority of participants (51/66 participants). Most individuals showed decreased levels of psychopathology severity, as well as a change in network domain scores, after one year, irrespective of symptom-network stability. We did not find evidence that the stability of contemporaneous individual symptom networks was associated with a change in psychopathology. Thus, our results do not support the hypothesis that psychopathological severity is directly linked to contemporaneous network structure.

Our results align with those presented by Snippe et al. [33], who found that treatment did not change the dynamic structure of symptom networks for individuals with depression, even though treatment did affect mean symptom levels. Although their conclusions were based on group-based multilevel analyses, which may have obscured individual differences linked to changes in symptom networks over time [34–37], our individual-centered approach led to similar conclusions. In our sample, participants also showed a significant decrease in mean levels of psychopathology and symptom domains, with most individuals showing no changes in symptom-network structure. While there was no statistical significant difference between absolute change scores of psychopathology between the stable and unstable group, there were some observations that we made. It appears that the stable group also shows more stability in the median scores on the diary domains than the unstable group, especially for stress, depression and confidence. However, due to the small sample size of the unstable group we were unable to reliably test whether these differences were statistically significant. Our results force us to reflect critically on the usefulness of network structure evaluations as a means to explain psychopathology severity. This is important to keep in mind in the development of intervention programs to improve mental health that work with feedback based on individual network structures (e.g., [13, 14, 38], as the link between (elements) of network structure and psychopathology severity or clinical state is not fully understood yet.

Adding to earlier work in this sample [20], we found that the edge between confidence and depression was present for the overlarge majority of participants at T0 as well as T1. This highlights the usefulness of individual symptom networks construction to gain more insight into how different domains of symptoms are connected, which in turn could provide useful input for research aimed at understanding the development of psychopathology. Especially in the case of confidence and depression, this highlights the potential relevance to incorporate constructs from positive psychology, e.g., increasing confidence as a protection mechanism to prevent the emergence or worsening of depressive symptoms [39].

Some limitations need to be taken into account when interpreting our results. First, several factors have limited the chance of finding a difference in psychopathology changes between the stable and unstable group. Group membership was based on INIT. As INIT tests similarity between individual network structures by comparing a model with equality constraints to a model in which all parameters have been estimated freely, this results in a large increase in the number of parameters estimated for the model without equality constraints. Hence, when it comes to detecting individual differences, INIT errs on the side of caution. Further applications could look into comparing multiple models to test intermediate forms of inequality in individual network structures over time. Second, the application of INIT resulted in a small number of individuals being allocated to the unstable group, which decreased the power to detect a statistical association between the stable and unstable group and changes in psychopathology. Moreover, as a statistical difference is not necessarily a *meaningful* difference, hypothetically, the difference between the stable and unstable group may have been smaller than anticipated, resulting in less discriminating groups than expected.

Furthermore, it is possible that important changes in individual network structures occur on a different level than modeled in this study. We used the most popular method to estimate individual network structures: the gVAR. The gVAR model assumes stationarity and so estimates deviations around an individual’s trend, rather than stable increases. We want to mention two points related to this. First, stationarity is a strong assumption that might not be tenable, especially in longer time-series. Second, idiographic network structures as estimated using gVAR show how temporal deviations from one variable’s mean affect another variable’s temporal deviations from its means. However, relations of the mean trends themselves are not part of the model, i.e., the model assumes one stable mean over time. Therefore, the extent to which mean changes in one variable influence mean changes in another variable, are currently not modelled, even though this change is arguably the most meaningful from a clinical (and experiential) perspective. To the extent that network theory describes patterns between means levels, our results are less informative for the theory, but highlights the importance of when and under what conditions network theory is expected to hold giving our current modeling tools. Incorporating changes in the mean of symptom (node) scores into the estimation of network structures is therefore an important future step that potentially shed light on the relationship between psychopathology and idiographic network structure.

One consideration is that in our network, we included five negative emotion nodes and one positive emotion node. We deliberately did not interpret whether network stability is positive or negative for each individual, rather, we investigate the association between network stability and psychopathology changes. In addition, our domain structure was chosen based on theoretical ground (i.e., which items typically cluster together clinically) rather than on factor analysis. A second consideration to keep in mind is that symptoms were measured only once a day, while the processes of symptom development may unfold within a shorter timeframe. In previous studies on the same dataset [20, 30], we observed that most relationships between variables appeared within days rather than over days. Therefore, we looked exclusively at contemporaneous relationships between variables, which due to the design of our study reflect the associations within a day. If the variables had been assessed on a different timescale, e.g., multiple measurements within a day, we might have found more temporal associations and we cannot exclude that this would have shown a different pattern of stability over time. One important difference between contemporaneous and temporal networks is that in the latter, the direction of associations can be estimated, and taking this directionality into account might lead to different results. For example, it is possible that the direction of the association changed (e.g., from depression → anxiety to anxiety → depression) between measurement occasions. This is something that temporal networks could detect, but contemporaneous networks could not. Thus, in our study, which is based on contemporaneous networks, we cannot make any conclusions about the stability of the directionality of associations. Therefore, this study should be replicated with measurements on different timescales to better understand potential temporal relations. In addition, it might be fruitful to explore alternative methodologies that explicitly account for the absence of temporal relationships.

A final limitation concerns the generalization to other samples and research settings. Replication in other samples that reflect other populations (e.g., the general population, individuals with depression), apply other designs (e.g., six-month diary periods, three assessments per day) and different operationalization of stability (e.g., density, centrality) is necessary to determine the extent to which our findings are specific to our study.

Nevertheless, our study is valuable for its unique design. Our convenience sample was designed as a diary study to predict course and outcome in individuals at different levels of risk for psychosis. In this sample, a change in psychopathology was expected, as, about 75% of participants in our sample received mental health care, which likely alleviates psychopathological severity over time. Therefore, it is a highly relevant sample to investigate the stability of symptom networks specifically in this group of individuals. It does seem likely to expect that, as these individuals received mental health care for some form of mental distress, that these interventions influenced levels of psychopathological severity. Clinically, our sample is quite diverse and although this is representative of those at UHR for psychosis, the heterogeneity in received care is also high. Unfortunately, we did not have detailed information on treatment and therefore could not take this into account in the analyses. In addition, data were collected over two separate diary periods spanning 90 days each, creating the unique opportunity to compare individual network models over time. With 90 days for each diary period, we were able to assess symptoms over a relatively long period, as many diary studies last only 5–7 days. This is an important advantage as more variation could be captured, and in turn, results are less dependent on the specific moment of the measurement period. In addition, with 180 measurements per individual for 66 individuals, we had a total of 11.880 data-points divided over two diary periods, creating the novel opportunity to relate network structures to changes in psychopathology within individuals along the early stages of the psychosis continuum.

## Conclusion

There is a pressing need to gain more understanding of the complexity of the development of psychopathology in individuals over time. Timely recognition is essential as this may prevent progression to more severe stages of mental illness. Estimating individual network models may offer interesting opportunities for clinical research and practice regarding early intervention, but is still in its early days. Our study is the first to directly examine the association between changes in individual network models and changes in psychopathology severity. The fact that we were unable to observe significant associations between changes in network structure and changes in psychopathology calls for further critical evaluation in order to optimize the implementation of clinical applications based on current methods. As such, this study can be viewed as a steppingstone on the road to further investigating the relationship between individual network structure and psychopathology.

## Supporting information

S1 File Preprocessing steps. (DOCX)

S2 File Sensitivity analyses. (DOCX)

S3 File Simulation study on using equality constrains to test network stability Set-up. (DOCX)

S1 Fig True network structure. (DOCX)

S2 Fig Simulation results for INIT when equality constraints are placed on unpruned networks. (DOCX)

S3 Fig Simulation results for INIT when equality constraints are placed on pruned network using a union model. (DOCX)

S1 Table Domains and corresponding items selected for analyses. (DOCX)
