# Supplementary material for: Relating stability of individual dynamical networks to change in psychopathology
Source: PLoS One. 2023 Nov 9;18(11):e0293200. doi: 10.1371/journal.pone.0293200 (PMC10635522; doi:10.1371/journal.pone.0293200)
Supplement: S1 File — (DOCX) [file pone.0293200.s001.docx]

**Supporting information 1: preprocessing steps**

**Missing data**. Individuals had on average 9% missing data points for Diary 1 (baseline) and 6 % missing data points for Diary 2 (1-year follow-up), ranging from 0 to 23%. Since there is no consensus in the literature on the best imputation strategy for imputing time series data, we tested six imputation strategies (MICE, Amelia, exponential moving average, linear moving average, Kalmal filter, and mean imputation) on our dataset (i.e., items used for analyses) in a previous study (van der Tuin et al. under review). For this, we used data from six individuals with complete data. First, we randomly deleted 5, 10 and 25 % of their data points. Next, for 1000 iterations we performed the six imputation strategies on the newly created datasets. As the real data values were known for these six individuals, we compared the newly imputed data points to the real values and averaged and squared the difference to calculate mean squared errors per imputation strategy. Exponential moving average performed best on our dataset and was therefore chosen as imputation strategy.

**Imputation**. There is not only no consensus in the literature on which imputation strategy should be used for time series data, but there is also research lacking on whether data should be imputed at all in these types of data. As a sensitivity analysis, we created symptom networks with and without imputations and compared the covariance matrixes within individuals using correlations. This showed relatively high correlations for both Diary 1 (range: .93 – 1.00, mean: .99) and Diary 2 (range: .61 – 1.00, mean: .99) and thus imputation did not influence results greatly. To ensure maximum power, we therefore decided to impute missing data.

**Normality assumption.** One of the assumptions of the ‘psychonetrics’ R-package, used to create the symptom networks, is that data is normally distributed. For some individuals and some items, data was heavily skewed. Therefore, data was transformed with a nonparanormal copula transformation (Lui et al. 2012) with the ‘huge’ package in R (Haoming et al. 2021). Previous research has shown that this form of transformation works well for heavily skewed data in symptom networks (Isvoranu & Epskamp, preprint; Mansueto et al. preprint).

**Stationarity.** Another assumption of the ‘psychonetrics’ R-package is that the data is trend-stationary. Plots showed a linear trend for most individuals and most items therefore domains were regressed against time before the main analyses and the residuals were entered in the graphical VAR model.
