# Supplementary material for: Relating stability of individual dynamical networks to change in psychopathology
Source: PLoS One. 2023 Nov 9;18(11):e0293200. doi: 10.1371/journal.pone.0293200 (PMC10635522; doi:10.1371/journal.pone.0293200)
Supplement: S2 File — (DOCX) [file pone.0293200.s002.docx]

**Supporting information 2: Sensitivity analyses**

Union model on pruned network models.

Using INIT, equality constraints can either be placed on unpruned networks, i.e., networks in which all edges are estimated to be non-zero, or on pruned networks, i.e., networks in which some edges have been shrunk to zero using a model selection procedure. This model selection procedure estimates sparse individual network structures with a high identification rate of the most substantial edges. In the analyses in the main paper, using INIT, equality constraints have been placed between the unpruned networks at T0 and T1 directly. As a sensitivity check, we have applied INIT on pruned network structures. Although INIT applied to pruned networks follow the same concept as when applied to unpruned networks, the application of INIT to pruned network structures requires some important additions to the basic technique. The procedure and results of INIT application to pruned network models are discussed in this appendix.

**Method.** INIT makes use of the psychonetrics package in R to estimate individual networks using Maximum Likelihood estimation (ML). After estimating the fully connected network, the network can be pruned using several pruning techniques. For our sensitivity analysis we choose to apply recursive pruning at an level of 0.05. This is a step-by-step procedure, i.e., in each iteration, non-significant edges are removed from the network, after which the remaining edges are re-estimated. Previous simulation studies have shown that GVAR estimation through ML combined with recursive pruning works well when estimating sparse individual network structures (Isvoranu & Epskamp, preprint; Mansueto et al., 2022).

Equality constraints are placed on the individual pruned networks at T0 and T1, using INIT. In order to place equality constraints between pruned network models INIT makes use of a union model. The union model will add all edge-weights to both networks at T0 and T1 that are estimated to be non-zero in at least one of the networks. A union model is a necessary step in the procedure because equality constraints cannot be placed directly between an edge that is estimated to be non-zero in one network and estimated to be zero in another. INIT follows the same basic logic as for the unpruned network case: an optimal model fit needs to be ensured such that the estimated network parameters of an individual at T0 are equal to their network structure at T1. When placing equality constraints on pruned networks, model fit is best evaluated with the BIC, see Appendix C.^^[[1]](#footnote-1)^^

Taking the Bayesian Information Criterion (BIC) as an indicator, the model fit of the network structures with equality constraints between T0 and T1 is compared to the model fit without imposing constraints on the network structures at T0 and T1. This results in either a better model fit for an equal network model over time, thus with constraints, or for a different network model over time, thus without constraints.

**Results.** Applying INIT on pruned network models showed that for 58 individuals out of 66, the model with equality constraints showed the best model fit according to the BIC. The other 8 individuals, the model without equality constraints fit better to the data. Applying INIT to pruned network models was more conservative in the number of differences estimated as 7 individuals who were placed in the “unstable” group would be labeled “stable” based on these results. Minor differences are to be expected as the BIC is more conservative. Further simulation studies need to be done in order to assess the exact number of differences between the two application procedures of INIT are to be expected.

Comparison of results INIT and correlation coefficients.

As an indication of the degree to which network parameter estimations of T0 and T1 resemble each other, we inspected Pearson correlation between the unconstrained unpruned network models at T0 and T1. In addition, we inspected this correlation for the stable and unstable group. Important to note here is that a high Pearson correlation reflects a strong linear relation between parameter estimates (i.e., differences in estimated edge weights at time point T1 can be predicted from differences at T0), but that a low Pearson correlation merely indicates the lack of such a linear relation; a low correlation may either indicate large differences between the networks at T0 and T1 or result from the fact that at one or both time points, all edges have approximately the same weight. As an additional sensitivity check, we compared the results from INIT to the correlation analysis.

The correlation between the edge weights of unpruned contemporaneous networks at T0 and T1 for the whole sample was, on average, equal to .66 (sd = .25, range: .09 - .93). When split, the mean correlation between unpruned contemporaneous networks at T0 and T1 was estimated at .69 (sd = .16, range: .09 - .93) in the stable group and .55 (sd = .17, range: .21 - .72) for the unstable group. While results from the INIT and correlation analysis generally align, this is not necessarily the case for each individual specifically, as shown by the large range of correlations observed across individuals. Further inspection of the correlations showed that for individuals from the stable group who had a low correlation, this was often due to a sparse network model. When many edges are set to zero, as is the case in a sparse network model, the covariance matrix has a small standard deviation (i.e., there are few differences between the parameter estimates), leading to a low correlation via a restriction of range effect; this can happen even though two networks are almost identical.

1. When placing equality constraints on pruned networks, the AIC turned out to be too liberal, meaning the AIC was in favor of the model without equality constraints even if the network structures were highly stable. The BIC adds a penalty for model complexity and therefore proved to be the best information criteria to guide decision regarding model equality in light of structural network differences when placing equality constraints on pruned network models. It is important to note here, due to the additional penalty to model complexity the BIC entails, using the INIT on pruned network models is likely to result in a more cautious estimation of the differences present. [↑](#footnote-ref-1)
