# Supplementary material for: Relating stability of individual dynamical networks to change in psychopathology
Source: PLoS One. 2023 Nov 9;18(11):e0293200. doi: 10.1371/journal.pone.0293200 (PMC10635522; doi:10.1371/journal.pone.0293200)
Supplement: S3 File — (DOCX) [file pone.0293200.s003.docx]

**Supporting information 3: Simulation study on using equality constrains to test network stability**

**Set-up**

To test the power to detect small differences in individual network structures using INIT we performed a small simulation study. INIT places equality constrains on the network model such that an optimal fit needs to be ensured in such a way that the network of an individual at a previous time point is equal to the network model at another time point. The fit of this model is then compared to the model fit of a network model in which the parameters of both network models for the individual are freely estimated.

[Figure B1 about here.]

For this simulation study we simulated data from a chain graph, see Figure B1, as this model is commonly used in simulation studies (Epskamp, 2018). We varied the number of time-points, the number of variables, and the amount of rewiring. Rewiring is taken as an indication of the number of differences between network models; where a rewiring of 0 means equal network models and a rewiring of 1 means all original edges are rewired, thus resulting in completely different network models. We varied the number of time points from t= 50 to t = 500 (t= 50, t = 100, t = 250, t =500), creating a range of plausible amount of time points to desirable amount of time points. Number of variables were 6 or 12, corresponding to the size of the network structure. We increased the amount of rewiring with steps of 0.1 from 0 to 1 (r = 0, r = 0.1, r = 0.2, r = 0.3, r = 0.5, r = 0.4, r = 0.5, r = 0.6, r = 0.7, r = 0.8, r = 0.9, r = 1). In addition, we preformed the test on unpruned network models as well as on pruned network models. We inspected the AIC rejection rate and the BIC rejection rate. The simulation study was performed in R (version 4.1.0).

Results

As can be seen in Figure B2, the AIC rejection rate is better to use when imposing equality constrains on unpruned network models, whereas the BIC rejection rate is better to use when imposing equality constrains when comparing pruned network models, see Figure B3. Furthermore, the test is sensitive to pick up even minor differences in network structure (rewiring is 0.1) when t = 100. Bigger differences (r = 0.2) are picked up when t = 50. The more differences, and the higher the number of time points, the more power INIT has to determine the presence of differences in the network structure.

[Figure B2 about here.]

[Figure B3 about here.]
