# Supplementary material for: Relating stability of individual dynamical networks to change in psychopathology
Source: PLoS One. 2023 Nov 9;18(11):e0293200. doi: 10.1371/journal.pone.0293200 (PMC10635522; doi:10.1371/journal.pone.0293200)
Supplement: S1 Fig — (DOCX) [file pone.0293200.s004.docx]

**S1 Fig. True network structure.** Network models were simulated to be chain graph. Rewiring is used to create different network structures for the different simulation conditions.
