# Supplementary material for: Relating stability of individual dynamical networks to change in psychopathology
Source: PLoS One. 2023 Nov 9;18(11):e0293200. doi: 10.1371/journal.pone.0293200 (PMC10635522; doi:10.1371/journal.pone.0293200)
Supplement: S2 Fig — (DOCX) [file pone.0293200.s005.docx]

**S2 Fig. Simulation results for the INIT when equality constraints are placed on unpruned networks.** We inspected the p-value, the BIC and the AIC. AIC performed best when placing equality constraints on unpruned networks.
