# Supplementary material for: Relating stability of individual dynamical networks to change in psychopathology
Source: PLoS One. 2023 Nov 9;18(11):e0293200. doi: 10.1371/journal.pone.0293200 (PMC10635522; doi:10.1371/journal.pone.0293200)
Supplement: S1 Table — (DOCX) [file pone.0293200.s007.docx]

**Supplementary Table 1**: **Domains and corresponding items selected for analyses.**

| **Domain** | **Item** |
| --- | --- |
| Irritation | Today I felt irritable  Today I felt irritated |
| Stress | How stressed were you today? |
| Depression | Today I felt down  Today I felt empty  Today I felt apathetic  I could experience pleasure when nice things happened today (reverse coded) |
| Psychosis (paranoia domain) | I felt suspicious today  Today I had the feeling that others disliked me  I felt that other could read my thoughts today  I felt unreal today  I felt that others could control me today |
| Anxiety | Today I felt anxious  I felt worried today |
| Confidence | I felt confident today  Today I could handle what came my way |
